# The Effect of Glycosylated Soy Protein Isolate on the Stability of Lutein and Their Interaction Characteristics

**DOI:** 10.3389/fnut.2022.887064

**Published:** 2022-05-24

**Authors:** Xia Wang, Shaojia Wang, Duoxia Xu, Jingwei Peng, Wei Gao, Yanping Cao

**Affiliations:** ^1^Beijing Advanced Innovation Center for Food Nutrition and Human Health (BTBU), School of Food and Health, Beijing Higher Institution Engineering Research Center of Food Additives and Ingredients, Beijing Technology and Business University (BTBU), Beijing, China; ^2^Chenguang Biotech Group Co., Ltd., Handan, China

**Keywords:** soy protein isolate, inulin-type fructans, lutein, stability, interaction

## Abstract

Lutein is a natural fat-soluble carotenoid with various physiological functions. However, its poor water solubility and stability restrict its application in functional foods. The present study sought to analyze the stability and interaction mechanism of the complex glycosylated soy protein isolate (SPI) prepared using SPI and inulin-type fructans and lutein. The results showed that glycosylation reduced the fluorescence intensity and surface hydrophobicity of SPI but improved the emulsification process and solubility. Fluorescence intensity and ultraviolet–visible (UV–Vis) absorption spectroscopy results showed that the fluorescence quenching of the glycosylated soybean protein isolate by lutein was static. Through thermodynamic parameter analysis, it was found that lutein and glycosylated SPI were bound spontaneously through hydrophobic interaction, and the binding stoichiometry was 1:1. The X-ray diffraction analysis results showed that lutein existed in the glycosylated soybean protein isolate in an amorphous form. The Fourier transform infrared spectroscopy analysis results revealed that lutein had no effect on the secondary structure of glycosylated soy protein isolate. Meanwhile, the combination of lutein and glycosylated SPI improved the water solubility of lutein and the stability of light and heat.

## Introduction

Lutein, an oxygen-containing fat-soluble carotenoid, is widely distributed in flowers, vegetables (1), fruits, egg yolks (2), algae, grains (3), etc. and is mainly derived from marigolds (4). Lutein is also present in the macular pigment of human eyes and plays an indispensable role in preventing eye diseases, such as age-related macular degeneration (AMD) (5). However, it cannot be synthesized within the human body and should be supplied from external sources, such as food (6). Lutein possesses a carbon skeleton and a polyolefin chain (7), a major chromophore group (8). The carbon chain is supported by hydroxyl-containing ionone rings on each side with three stereocenters, β-ionone ring containing a stereo center (3R), and ε-ionone ring containing two stereocenters (3′R and 6′R) (9). The eight conjugated olefins in the carbon chain of the lutein molecule make lutein susceptible to degradation by the external environment (10).

The encapsulation systems, including liposomes, emulsions, gels, and molecular complexes, are often used to inhibit the degradation and improve the stability of biologically active substances, such as lutein (11). Of them, the macromolecules' cavity (such as protein) has attracted increasing interest in the encapsulation of biologically active substances. It is reported that egg white albumin improves the stability of marigold lutein ester extract during storage, and lutein dipalmitate binds spontaneously to egg protein through van der Waals forces and hydrogen bonding (12). Yi et al. (13) found that the stability of lutein increased with the increase of milk protein content, the protective effect of sodium caseinate (SC) on lutein was stronger than the whey protein isolate (WPI), and the milk protein interacted with lutein through hydrophobic bond.

Soy protein isolate (SPI) is a plant protein (about 90% protein) with high nutritional value and is widely used in the food industry (14, 15). It is mainly composed of β-conglycinin (7S, 180–210 kDa) and glycinin (11S, approximately 360 kDa) and accounts for about 70% of the total protein. The 11S component consists of 6 acidic and 6 basic polypeptide chains linked together by disulfide bonds, whereas the 7S is stabilized by hydrophobic interactions (16). In recent years, SPI has been used as a carrier in food-grade delivery systems to improve the stability of biologically active substances. Cao et al. (17) found that SPI could improve the thermal stability of chlorophyll. Tapal and Tiku (18) improved water solubility and bioavailability of curcumin using SPI. Wan et al. (19) also showed that SPI could be used as a carrier of resveratrol (RES) in functional foods, which could improve the water solubility and stability of RES. However, the functional properties of SPI, such as solubility in neutral and mildly acidic environments, severely limit its use as an encapsulation wall material. As such, the physical, chemical, and enzymatic properties of SPI are often modified (16). Physical modification mainly relies on high temperature and high pressure or shearing, making the equipment requirements relatively high and large-scale production difficult. Enzymatic modification is expensive, and protein hydrolysis can make the taste worse. Chemical modification has a high effect on the functional properties of the protein, mainly by introducing food-grade ingredients (20). Glycosylation is a typical chemical modification, which modifies the structure of the protein and improves the functional properties of the protein through a covalent bond between the amino groups of protein and the carbonyl group of the reducing sugar (21). The glycosylation of glucan and protein improves the freeze–thaw stability of SPI hydrolysate (SPIH) emulsion (22), the binding and transporting ability of casein phosphopeptide to calcium (23), the solubility, emulsifying, and foaming ability of peanut protein isolate (24). The emulsifying properties and emulsifying stability of the conjugates of inulin and WPI were significantly higher than the WPI at a pH of 3–7 (25). Inulin-type fructans (ITFs) are linear fructose polymers with predominantly or only β-(2 → 1) fructosyl–fructose linkages (26). Stabilizing the intestinal mucosal barrier, reducing the risk of colon cancer, promoting calcium absorption, and improving constipation are the physiological functions of ITFs (27–33). ITFs are widely used as a prebiotic food ingredient (34). However, the modification effects of ITFs on SPI and ITF-modified SPI on the stability of lutein are still unknown.

Therefore, the present study aimed to study the modification effect of ITFs on SPI and ITF-modified SPI (glycosylated soybean protein isolate, GSPI) on the stability of lutein and the interaction between GSPI and lutein. The study results will provide insights into the preparation of protein-encapsulating systems for embedding lutein and other similar biologically active compounds.

## Materials and Methods

### Materials

Soy protein isolate was purchased from Shanghai Yuanye Bio-Technology Co., Ltd. (Shanghai, China). ITFs and lutein extract were provided by Chenguang Biotech Group Co., Ltd. (Hebei, China). Phosphates were purchased from Shanghai Aladdin Biochemical Technology Co., Ltd. (Shanghai, China). Lutein (97%) was purchased from ChromaDex, USA. Bovine serum albumin (BSA, purity 97%) was obtained from Beijing Solarbio Science and Technology Co., Ltd. (Beijing, China). Sodium 8-naphthalenesulfonate-1-anilino (ANS) ≥97% was purchased from Shanghai Macklin Biochemical Technology Co., Ltd. (Shanghai, China). G250 Coomassie Brilliant Blue Ultra pure grade was purchased from Beijing Boao Tuoda Science and Technology Co., Ltd.

Absolute ethanol (chromatographic grade), methyl tert-butyl ether (chromatographic grade), methanol (chromatographic grade), cyclohexane (chromatographic grade), and N-hexane (chromatographic grade) were purchased from Beijing Merida Technology Co., Ltd. (Beijing, China). Ethyl acetate (analytical grade) and potassium hydroxide (KOH) (analytical grade) were purchased from Tianjin Fuchen Chemical Reagent Company (Tianjin, China). 2, 6-Di-tert-butyl-*p*-cresol (BHT) (analytical grade) was purchased from Shanghai Macklin Biochemical Technology Co., Ltd. (Shanghai, China).

### Sample Preparation

Soy protein isolate and ITFs (mass ratio 1:1) were stirred overnight with 10 mM Na_2_HPO_4_ to make them fully hydrated and adjusted to pH 11 with 0.1 M NaOH to make the final protein concentration of 2% (w/v). The SPI/ITF mixtures were incubated in a water bath at 100°C for 4.7 h (previous research work has confirmed that the GSPI has the highest emulsification ability under this experimental condition). HSPI was the only heated SPI (100°C for 4.7 h), and G-SPI was the physical mixture of SPI and ITFs.

Lutein extract was dissolved in absolute ethanol and stored at 4°C protecting from light. The lutein content in the lutein ethanol solution was expressed as 10 mg/ml with 97% lutein standard product.

For stable analysis of protein and lutein complexes preparation, 15 ml of GSPI and SPI were adjusted to pH 7.0, and then 0.5 ml of lutein extract in absolute ethanol was added dropwise to GSPI and SPI, respectively. Finally, the volume was adjusted to 100 ml with 10 mM pH 7.0 phosphate-buffered saline (PBS) and incubated for 1 h at room temperature.

The complexes of different concentrations of 97% lutein (10, 20, 30, 40, 50, 60, and 70 μM) and GSPI (0.2 mg/ml) were also prepared to explore the interaction between lutein and protein.

### Fourier Transform Infrared Spectroscopy

The freeze-dried sample was mixed with KBr at a ratio of 1:100 with KBr as the background. The wave number was 400–4,000 cm^−1^, and the resolution was 4 cm^−1^ for 32 scans with IS5 infrared spectrometer (Thermo Fisher, USA).

The spectral range of 1,700–1,600 cm^−1^ (amide I) of GSPI and GSPI–lutein were subjected to Fourier deconvolution and second derivative analysis by PeakFit 4.12.

### Fluorescence Spectrum

Later, 200 μl of all the samples were transferred to 96-well plates for fluorescence spectroscopy, and the intrinsic fluorescence spectrum was measured by M200 Pro TECAN Infinite multifunctional microplate reader (Tecan Inc., Switzerland).

#### Excitation Spectrum

The fluorescence excitation spectra of SPI, HSPI, G-SPI, and GSPI were measured at 293 K. The excitation wavelength was set from 250 to 380 nm and the emission wavelength was 420 nm (25).

#### Emission Spectrum

The fluorescence emission spectra of SPI, HSPI, G-SPI, and GSPI were measured at 293 K, and the fluorescence emission spectra of GSPI (0.2 mg/ml) and lutein (10, 20, 30, 40, 50, 60, and 70 μM) were measured at 293, 303, and 313 K. The emission wavelength was set from 310 to 460 nm, and the excitation wavelength was set at 280 nm, according to the method of Qi et al. (35).

The fluorescence quenching mechanism between GSPI and lutein was explored by the Stern–Volmer equation (1) (17):


(1) $$\begin{array}{l} \frac{F_{0}}{F}\;=\;1\;+K_{SV}\times \;\left[Q\right]\;=\;1\;+K_{q}\times \tau_{0}\times \;\left[Q\right] \end{array}$$

where, *F*_0_ and *F* are the fluorescence intensity in the absence and presence of lutein. K_SV_ is the Stern–Volmer quenching constant. K_q_ is the bimolecular quenching rate constant. τ_0_ is the average lifetime of the unquenched fluorophore, which is 10^−8^ s. [Q] is the concentration of lutein.

The double logarithmic Stern–Volmer equation was used to analyze the binding sites and binding constants of GSPI and lutein [Equation (2)]. The thermodynamic parameters of GSPI and lutein were determined by Equations (3, 4).


(2) $$\begin{array}{l} \log\frac{\left(F_{0}-F\right)}{F}\;=\;log\;K_{a}+n\;log\left[Q\right] \end{array}$$

where, *n* is the number of binding sites. K_a_ is the binding constant.


(3) $$\begin{array}{l} Ln\;K_{a}\;=\;-\frac{\Delta H}{RT}+\frac{\Delta S}{R} \end{array}$$

(4) $$\begin{array}{l} \Delta G\;=\;\Delta H-T\Delta S\;=\;-RT\;Ln\;K_{a} \end{array}$$

where, ΔH denotes the enthalpy change, ΔG denotes free energy change, and ΔS denotes entropy change. R denotes the gas constant 8.314 J/ (K mol). *T* (K) refers to different temperatures (20°C, 30°C, and 40°C).

### Surface Hydrophobicity

The 1-anilino-8-naphthalenesulfonate (ANS) was used as a fluorescent probe to measure the surface hydrophobicity (H_0_), according to the method of He et al. (20), with slight modifications. The sample was diluted with 10 mM pH 7.0 PBS to 0.01–0.09 mg/ml, then 2 ml of the protein sample was added with 40 μl ANS, and reacted for 10 min in the dark. Approximately 200 μl of each sample was transferred to a 96-well plate, and the fluorescence intensity was measured at 390 nm (excitation wavelength) and 470 nm (emission wavelength) using M200 Pro TECAN Infinite multifunctional microplate reader. The slope of the protein concentration and its corresponding fluorescence intensity were H_0_.

### Solubility and Emulsification

#### Solubility

The samples were centrifuged at 4,500 r/min for 15 min using an Avanti JXN-30 floor-standing centrifuge (Beckman, USA). The supernatant was diluted by 200 times, then 1 ml of the diluted solution was added with 5 ml of G250 Coomassie Brilliant Blue, mixed uniformly, and the absorbance was measured at 595 nm using an UVmini-1240 UV Spectrophotometer (Shimadzu, Japan). The samples were measured within 2–30 min. BSA was used as the standard curve to calculate the protein content.

#### Emulsification

Approximately 40 ml of GSPI and 10 ml of soybean oil were put into a 100-ml beaker, homogenized at a speed of 12,000 r/min for 2 min using T25 digital homogenizing and dispersing instrument (IKA, Germany) (36). Then, 50 μl was taken from the bottom and mixed with 5 ml of 0.1% SDS, and the absorbance values were measured at 500 nm. Afterward, 50 μl of deionized water and 5 ml of 0.1% SDS were adjusted to zero. The emulsifying activity index (EAI) was calculated by Equation (5).


(5) $$\begin{array}{l} EAI\;\left(m^{2}/g\right)=\frac{2\cdot 2.303\cdot A\cdot n}{C\cdot \left(1-“\varphi^{\prime \prime }\right)\cdot 10^{4}} \end{array}$$

where, A is the absorbance value at 500 nm, *n* is the dilution factor, *C* is the protein concentration (g/ml), and ϕ is the oil phase volume.

### UV–Vis Absorption Spectroscopy

The mixtures of GSPI and lutein in 2.2 were diluted four times, namely GSPI (0.025 mg/ml) with lutein standards (2.5, 5.0, 7.5, 10, and 12.5 μM). The ultraviolet–visible (UV–Vis) spectra were measured in the wavelength range of 190–350 nm using a 10-mm quartz cell with TU-1900 Dual-Beam UV–Visible Spectrophotometer (Beijing Puxi General Instrument Co., Ltd., China.).

### X-Ray Diffraction

The crystal of lutein, GSPI, and GSPI–utein was measured by a D8 Advance X-ray Diffractometer (Bruker Technology Co., Ltd., Germany) at a speed of approximately 2°/min and scanning at an angle (2θ) from 5° to 40°.

### Stability of Lutein

Thermal stability: Around 8 ml of lutein, SPI–lutein, and GSPI–lutein were added into a pressure test tube, tightened the tube mouth, and then heated in an oil bath at 120°C for 50 min. The samples were taken every 10 min.

Light stability: Lutein, SPI–lutein, and GSPI–lutein were placed in a 100-ml sample bottle, irradiated under a 302-nm UV lamp for 12 h, and the samples were taken every 2 h for analysis. (The light was perpendicular to the sample, and the distance was about 2 cm.)

#### Color Measurements

The color characteristics were measured by a CR-800 Spectrophotometer under a reflection mode (Beijing Colorimeter Instrument Equipment Co., Ltd., China). The total color difference (ΔE) was calculated by Equation (6):


(6) $$\begin{array}{l} \Delta E=\sqrt{{\left(L^{*}-L_{0}^{*}\right)}^{2}+{\left(a^{*}-a_{0}^{*}\right)}^{2}+{\left(b^{*}-b_{0}^{*}\right)}^{2}} \end{array}$$

where, ΔE means total color difference. ${\text{L}}_{0}^{*}$, ${\text{a}}_{0}^{*}$, and ${\text{b}}_{0}^{*}$ mean the initial color values of the sample. L^*^, a^*^, and b^*^ mean the color values of the sample at time *t*. L^*^ means light and dark; a^*^ means red and green, and b^*^ means yellow and blue.

#### Content and Degradation Kinetics Analysis

Around 5 ml of the above sample was used for stability analysis. Then, 10 ml of absolute ethanol was mixed with 10 ml of 60% KOH, then shaken at room temperature for 3 h, and extracted with cyclohexane:*n*-hexane:ethyl acetate = 1:2:2. The extraction was repeated 3 times. The extracts were combined and rotary evaporated to near dryness in a 30°C water bath, then dissolved to 10 ml using a 0.1% BHT absolute ethanol for HPLC analysis. A standard curve was plotted using 97% lutein to characterize the content changes of lutein during the degradation process.

The change in lutein content was determined by Waters 2,695 HPLC (Waters Technology Co., Ltd., USA). The chromatographic column model was Venusil XBP C30 (5 μm, 250 mm × 4.6 mm). Phase A (methanol:water = 88:12), phase B (methyl tert-butyl ether). At 0–18 min, phase A changed from 100% to 10%; at 18.1 min, phase A changed from 10% to 100%, and kept it for 10 min. The flow rate was 1.0 ml/min, and the injection volume was 50 μl. The degradation rate was calculated by Equation (7), and the degradation kinetics was determined by Equations (8–10).


(7) $$\begin{array}{l} Degradation\;rate\;=\;\frac{C_{0}-C_{t}}{C_{0}}100\% \end{array}$$

Kinetic equations (8–10) (37)


(8) $$\begin{array}{l} C_{0}C_{t}\;=\;k_{0}t \end{array}$$

(9) $$\begin{array}{l} Ln\;\left(C_{t}/C_{0}\right)\;=\;k_{1}t \end{array}$$

(10) $$\begin{array}{l} \frac{1}{C_{t}}\;-\;\frac{1}{C_{0}}\;=\;k_{2}t \end{array}$$

where, *C*_0_ represents the initial content of lutein before stability analysis, and *C*_t_ represents lutein content at time *t* during stability analysis. k_0_, k_1_, and k_2_ represent the kinetic constants.

### Size Distribution

The particle size of lutein, GSPI, and GSPI–lutein was measured by an S3500 laser particle size analyzer (Microtrac, Germany). The sample was added dropwise to the sample cell until the screen showed ready, and then proceeded to the particle size distribution measurement.

### Statistical Analysis

The images were generated by Origin 2021 software. The data were analyzed by one-way analysis of variance using IBM SPSS statistics 26. The significance analysis was done by LSD and Duncan's test (*p* < 0.05).

## Results and Discussion

### Effect of Glycosylation on Protein Properties

#### Fourier Transform Infrared Spectroscopy

**Figure 1:**
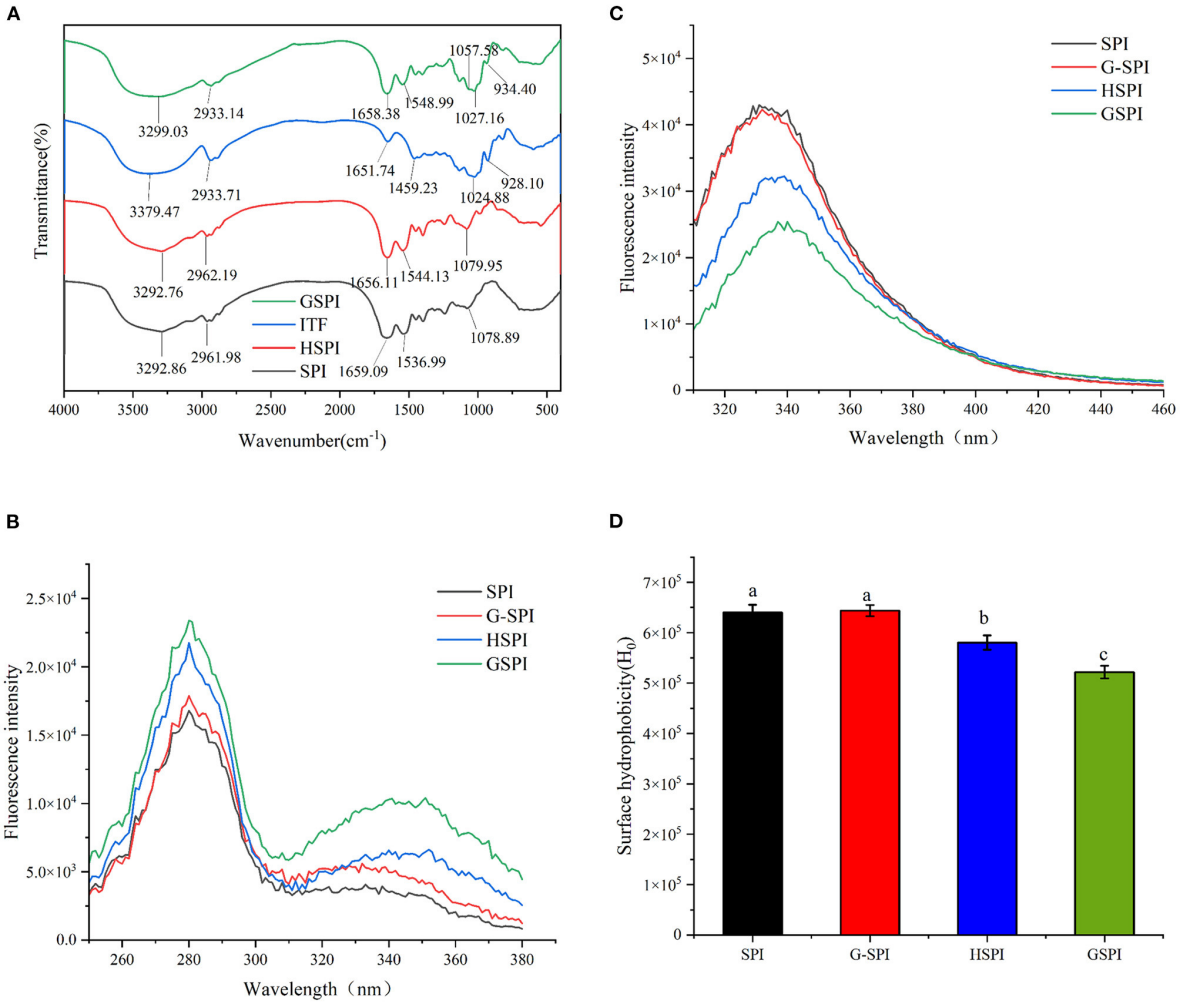
**(A)** The Fourier transform infrared spectroscopy of SPI, HSPI, ITFs, and GSPI. **(B)** Fluorescence excitation spectroscopy of 0.1 mg/ml SPI, HSPI, G-SPI, GSPI, *T* = 293 K, pH = 7.0, λ_ex_ = 280 nm, and λ_em_ = 310–460 nm. **(C)** Fluorescence emission spectroscopy of 0.1 mg/ml SPI, HSPI, G-SPI, GSPI, *T* = 293 K, pH = 7.0, λ_em_ = 420 nm, and λ_ex_ = 250–380 nm. **(D)** Surface hydrophobicity of 0.01–0.09 mg/ml SPI, HSPI, G-SPI, GSPI, *T* = 293 K, pH = 7.0, λ_em_ = 470 nm, and λ_ex_ = 390 nm. Different letters indicate significant differences.

Fourier transform infrared (FTIR) could determine the occurrence of glycosylation reaction. The amide I bands (1,700–1,600 cm^−1^) and amide II bands (1,600–1,500 cm^−1^) of protein are the most sensitive regions related to protein conformation (38). The C=O stretching vibration of the peptide bond, the C–N stretching vibration of the amino group, and the N–H bending of the amino group are the characteristics of this amide zone (39). Figure 1A depicts the FTIR results of SPI, HSPI, ITFs, and GSPI under the range of 4,000–400 cm^−1^. The amide I region of the GSPI absorption peak shifted from 1659.09 to 1658.38 cm^−1^, and the absorption intensity was slightly reduced, which might be due to the decrease in the carbonyl content. The Schiff base and pyrazine formed during glycosylation showed an absorption at 1658.38 cm^−1^ (23). The amide II band shifted from 1536.99 to 1548.99 cm^−1^, and the absorption peak of GSPI was stronger than SPI, indicating that the glycosylation product was formed by covalent bonds. This result was consistent with the results of glycosylated modified egg white protein pretreated by ball milling (40). The absorption intensity of GSPI was higher than SPI at the wavenumber of 1057.58 cm^−1^, indicating that GSPI produces a new C–N covalent bond, which was consistent with the findings of WPI and inulin (25). Additionally, there was a broad stretching vibration peak at 3,700–3,200 cm^−1^, intimating the existence of hydrogen bonds and the increase of free hydroxyl content. This result was consistent with the Maillard reaction products of whey protein and flaxseed gum (41).

#### Fluorescence Intensity

Glycosylation is a chemical method for modifying proteins by covalent bonding between the proteins and sugars (40). The fluorescent substances with characteristic peaks between 340 and 370 nm are produced during a reaction (42). As depicted in Figure 1B, SPI, HSPI, G-SPI, and GSPI had a characteristic protein excitation wavelength at 280 nm, whereas GSPI had another maximum excitation wavelength at 351 nm. These results confirmed the occurrence of the Maillard reaction and the production of fluorescent substances. This was consistent with the previous research result showing that WPI and inulin had a maximum fluorescence excitation wavelength of 344 nm (25). Figure 1C depicts the fluorescence emission spectrum, indicating that the fluorescence intensity of SPI and G-SPI had not much difference. The fluorescence intensity of GSPI was less than SPI, suggesting that ITFs had a shielding effect on the fluorescence of SPI. Some previous studies had also manifested that the fluorescence intensity of glycosylated protein was less than the original protein (43), and this effect increased with the decrease of dextran molecular weight. Notably, heating increased the fluorescence intensity of the protein, and the fluorescence intensity of WPI increased by dry heat treatment (44). However, in this study, the fluorescence intensity of HSPI was less than SPI. Therefore, it was speculated that this phenomenon was caused by extreme pH. Zhao et al. (45) revealed that extreme alkaline pH treatment of PSE-like chicken proteins had the same result.

#### Surface Hydrophobicity

Surface hydrophobicity is one of the essential functional properties of the protein, reflecting protein conformation and structure (46). ANS is commonly used as a probe to determine H_0_ of proteins, which binds to the hydrophobicity of proteins efficiently (47). Figure 1D depicts the change in H_0_ of SPI, G-SPI, HSPI, and GSPI, indicating that the physical mixture of SPI and ITFs was not significantly different from the H_0_ of SPI. Compared with SPI, the H_0_ of HSPI was significantly lower, which might be due to the fact that the alkaline conditions could make the protein fold and reduce the exposure of the SPI hydrophobic group. The H_0_ decreased significantly after glycosylation and the exposed hydrophobic groups of SPI bound to ITFs, resulting in a decrease in H_0_. In previous studies, the same results were obtained after glycosylation of SPI with *Pleurotus eryngii* polysaccharide (PEP) or dextran (DX) (20, 43). H_0_ was consistent with the fluorescence emission spectroscopy results, and similar results were obtained under extremely acidic pH excursions and mild heating conditions for SPI (48).

#### Emulsification and Solubility

**Figure 2:**
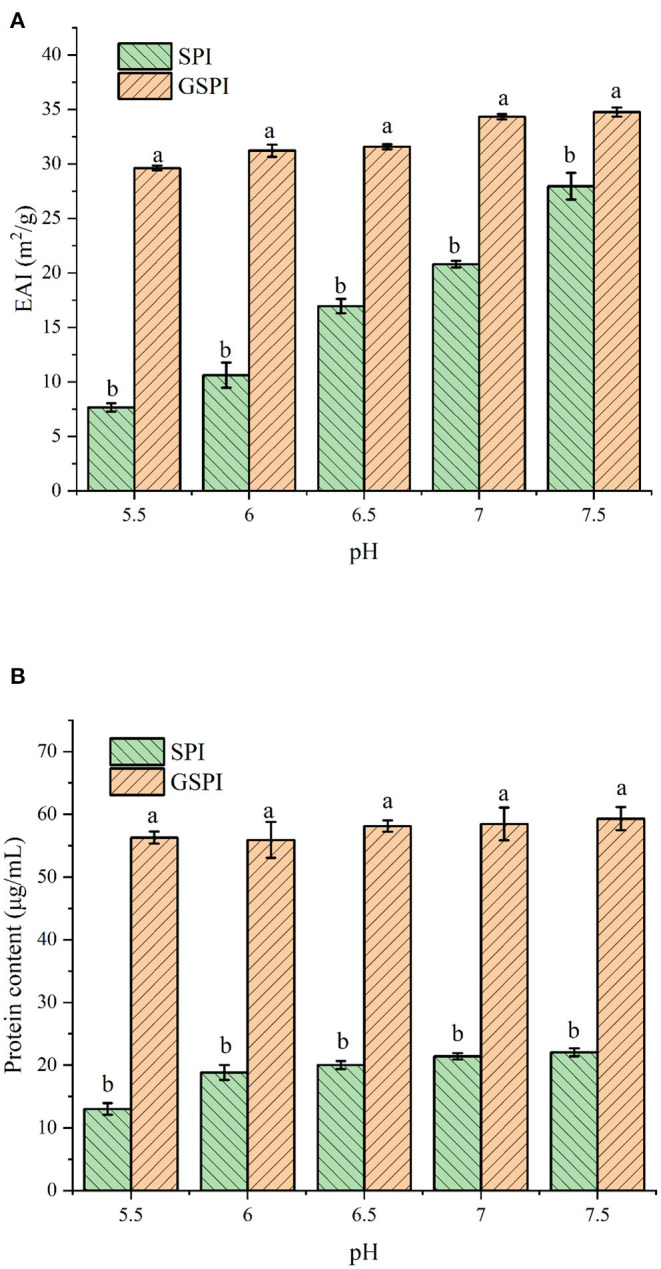
**(A)** Emulsification of SPI and GSPI at pH 5.5–7.5. **(B)** Solubility of SPI and GSPI at pH 5.5–7.5. Different letters indicate significant differences between SPI and GSPI at the same pH.

The EAI of protein is involved in several parameters, such as solubility, hydrophobicity, and structural flexibility (49). EAI is often used to indicate the emulsification of protein. As shown in Figure 2A, The EAI of SPI and GSPI were measured at different pH conditions, and the results showed that the emulsifying ability of GSPI was greater than SPI under the pH range of 5.5–7.5. Compared with GSPI, the EAI values of SPI were much more affected by pH. The poor solubility of SPI limits its application. The solubility of glycosylation significantly increased, and the protein content of GSPI diluted by centrifugation was 56.29 ± 0.96–59.30 ± 1.85 μg/ml, with no significant change in pH 5.5–7.5, whereas SPI had poor solubility in neutral and weakly acidic conditions (Figure 2B).

### Interaction Between GSPI and Lutein

#### Fluorescence Quenching

**Figure 3:**
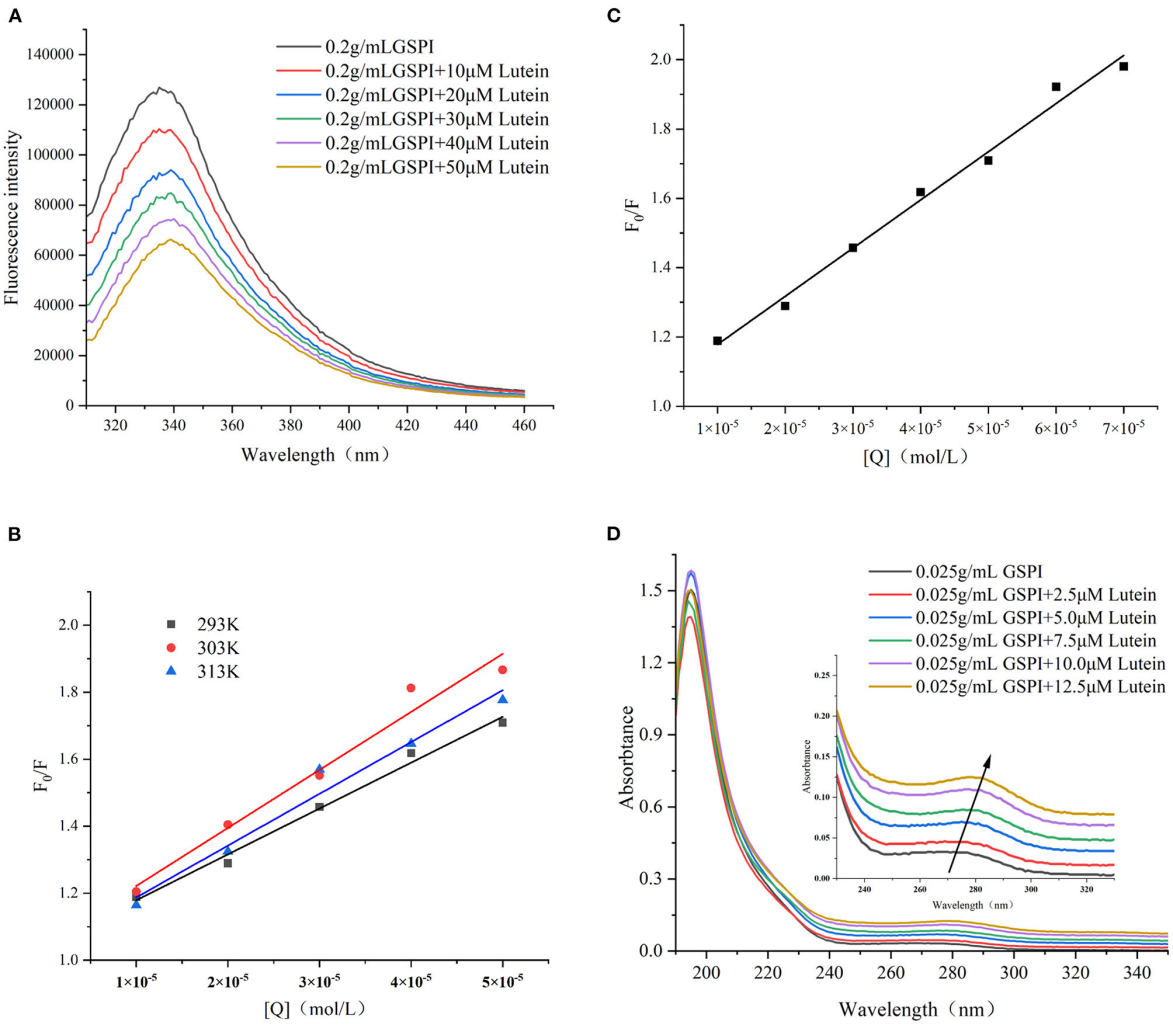
**(A)** Fluorescence spectra of GSPI (0.2 mg/ml) with different concentrations of lutein (0–50 μM), *T* = 293 K, pH = 7.0, and λ_ex_ = 280 nm. **(B)** The linear Stern–Volmer plot for lutein binding to GSPI at 293, 303, and 313 K. **(C)** The Stern–Volmer plot for lutein binding to GSPI with lutein (0–70 μM) at 293 K. **(D)** UV–Vis spectra of GSPI (0.2 mg/L) with different concentrations of lutein (0–50 μM), *T* = 293 K, and pH = 7.0.

Fluorescence quenching methods could reveal the protein conformation and/or dynamic changes in the macromolecular systems. The Stern–Volmer equation is the easiest method to determine the quenching pattern (50). Quenching occurs when the quencher is located near or in contact with the fluorophore (51). Protein fluorescence arises from the presence of multiple fluorophores, mainly including three aromatic amino acids—Phe, Tyr, and Trp (52). The changes in the amino acid environment change the fluorescence intensity. As depicted in Figure 3A, with the increase of lutein (10, 20, 30, 40, and 50 μM), the fluorescence intensity of GSPI continued to decrease, accompanied by a red shift, indicating an interaction between lutein and GSPI, which changed the microenvironment of the fluorophore (53).

Fluorescence quenching includes static quenching, dynamic quenching, and combined static and dynamic quenching. Static quenching is caused by the formation of complexes, whereas dynamic quenching is caused by intermolecular collisions. The dynamic and static combination quenching is caused by the formation of complexes and intermolecular collisions. As shown in Figure 3B and Table 1, the slope (K_SV_) of 303 and 313 K was >293 K, indicating that the fluorescence quenching of GSPI was caused by the collision of lutein and GSPI, which was a sign of dynamic quenching. However, the value of K_q_ far exceeded the maximum value of the dynamic quenching rate constant [10^10^/(mol s)], indicating that the quenching was static. Thus, it could not be concluded if it is a static or dynamic quenching.

**Table 1:** Lutein induces the Stern–Volmer quenching constant (K_SV_) and bimolecular quenching rate (K_q_) of GSPI at different temperatures (293, 303, and 313 K).

***T* (K)**	**K_**SV**_ (10^**4**^/mol)**	**K_**q**_ (10^**12**^/(mol s))**	***R* ^2^**
293	1.3689	1.3689	0.9899
303	1.7122	1.7122	0.9739
313	1.5495	2.7171	0.9715

The quenching effects of high lutein concentration on GSPI fluorescence and UV–Vis were studied to evaluate the type of quenching between GSPI and lutein. In the case of dynamic quenching, the relationship between *F*_0_/*F* and high-lutein concentration might concave toward the *Y*-axis (54). Figure 3C depicts a good linear relationship between increasing lutein concentration and *F*_0_/*F* (*R*^2^ = 0.9901), with no concave to the *Y*-axis (55). It also confirmed that there was no dynamic quenching between lutein and GSPI.

Ultraviolet–visible is a simple and effective method for the interaction between small molecules and proteins (56). Dynamic quenching does not change the absorption spectrum of the fluorophore, whereas complex formation (static quenching) changes the absorption spectrum of the fluorophore (57, 58). As depicted in Figure 3D, with the addition of lutein, the absorption intensity of the ultraviolet spectrum increased, which was accompanied by a significant red shift (Δλ = 10 nm). This indicated that lutein formed a complex with GSPI (59), leading to a red shift in the spectrum (60, 61). Therefore, lutein quenched the fluorescence of GSPI by static quenching.

#### Binding Forces and Thermodynamic Parameters

**Figure 4:**
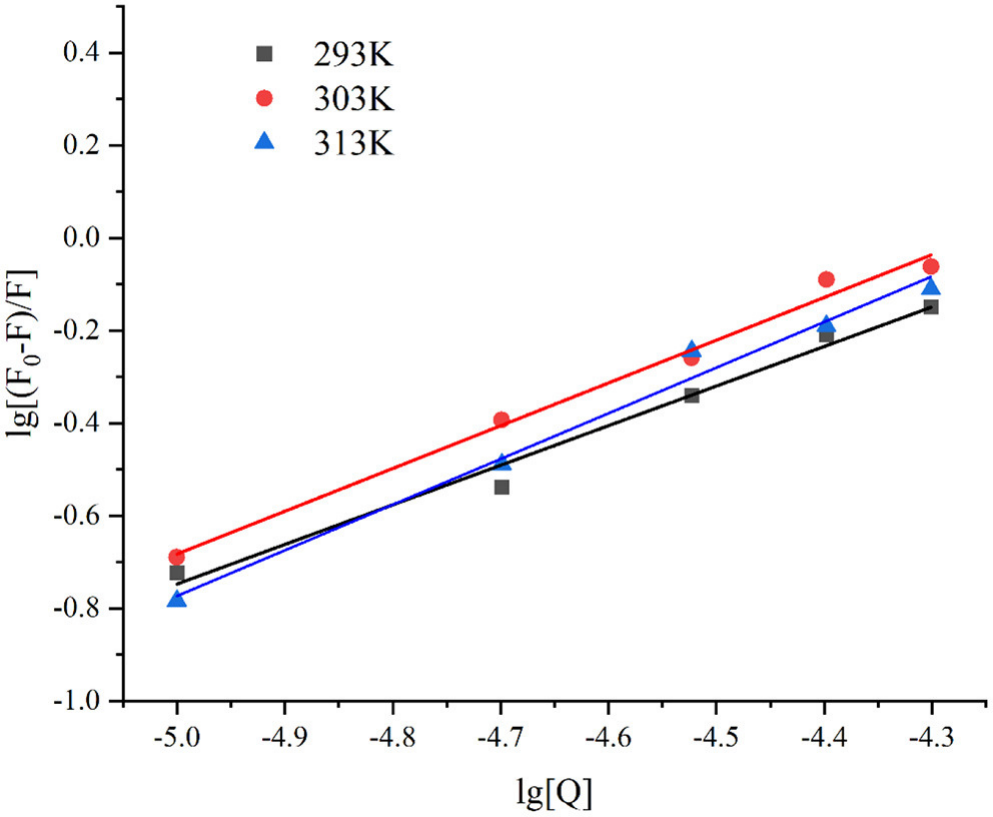
The linear plot of lg [(*F*_0_-*F*)/*F*] against lg [Q] at different temperatures, *T* = 293, 303, and 313 K.

The binding constant (K_a_ value) reflects the strength of the binding force (62). As depicted in Figure 4, the lg [(*F*_0_-*F*)/*F*] and lg[Q] presented a good linear relationship (*R*^2^ > 0.98). The *n*-values of the three different temperatures at 293, 303, and 313 K were 0.8551, 0.9237, and 0.9861, respectively, indicating that there was only one binding site. The K_a_ values of lutein and GSPI interaction at 293, 303, and 313 K showed an increasing trend with the increase in temperature (Table 2), suggesting that the GSPI–lutein complex was relatively stable at higher temperatures (63).

**Table 2:** Binding parameters and thermodynamic parameters of GSPI–lutein system at different temperatures.

**T (K)**	**K_**a**_ (10^**4**^ L /mol)**	**n**	***R* ^2^**	**ΔH (kJ mol^**−1**^)**	**ΔS (kJ mol^**−1**^)**	**ΔG (kJ mol^**−1**^)**
293	0.3189	0.8551	0.9846	50.64	241.79	−20.09
303	0.8701	0.9237	0.9908			−22.85
313	1.4315	0.9861	0.9854			−24.91

The thermodynamic parameters, such as ΔG, ΔH, and ΔS, could reveal the GSPI–lutein interaction (64). The value and sign of ΔH and ΔS could reflect the interaction force between small molecules and proteins (65). The forces between the biologically active substances and proteins mainly include hydrophobic interaction, hydrogen bonding, van der Waals force, electrostatic attraction, etc. (66). The interaction force could be divided into four types: for ΔH > 0 and ΔS > 0, the main force is hydrophobic interaction; for ΔH > 0 and ΔS < 0, the main force is electrostatic and hydrophobic interaction; for ΔH < 0 and ΔS < 0, hydrogen bonding and van der Waals force play a major role; and for ΔH < 0 and ΔS > 0, electrostatic interaction plays a principal role (67). As summarized in Table 2, ΔH > 0 and ΔS > 0 demonstrated that the interaction force between lutein and GSPI was mainly hydrophobic interaction. Yi et al. (13) reported similar results for WPI and SC (13). Furthermore, the values of ΔG (−20.09, −22.85, and −24.91 kJ mol^−1^) were negative at 293, 303, and 313 K, indicating spontaneous binding of lutein with GSPI.

#### X-Ray Diffraction

**Figure 5:**
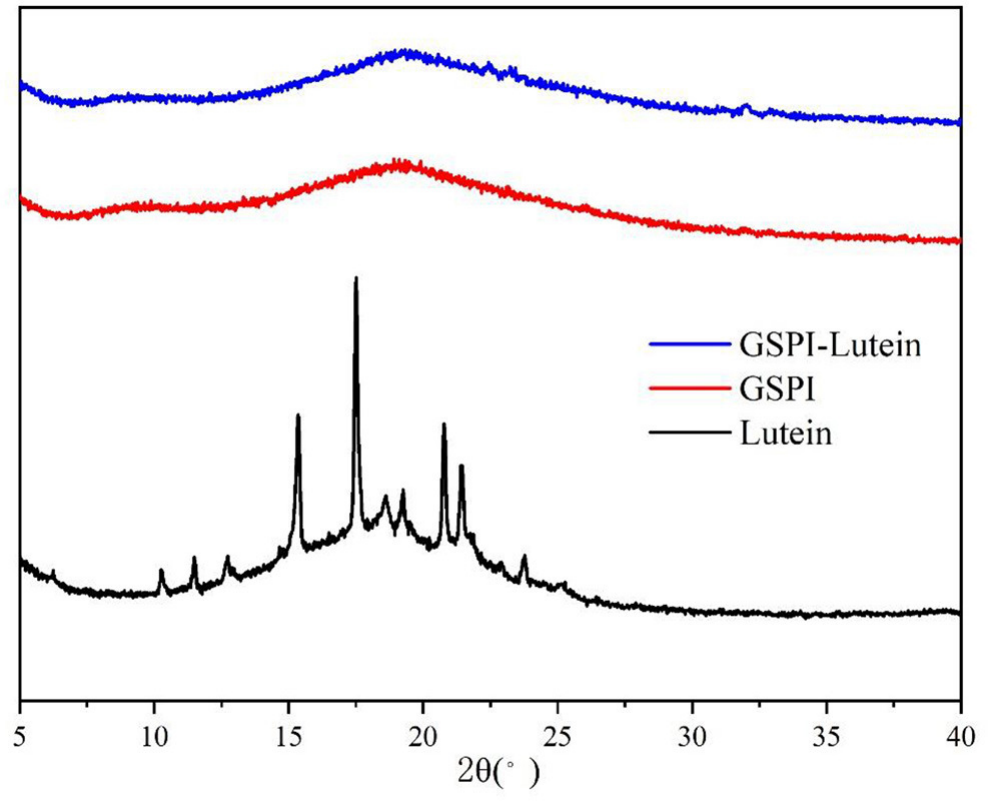
X-ray diffraction of lutein, GSPI, and GSPI–lutein.

The crystal analysis of the sample was performed by XRD. Figure 5 depicts the diffraction patterns of lutein, GSPI, and GSPI–lutein. Lutein showed a strong diffraction peak within 10°-25° (2θ), suggesting that lutein had a highly crystalline structure. After the combination of lutein and GSPI, the frontal characteristic diffraction peak of lutein did not appear, showing the presence of lutein in GSPI in an amorphous form (68).

#### Secondary Structure Analysis

**Figure 6:**
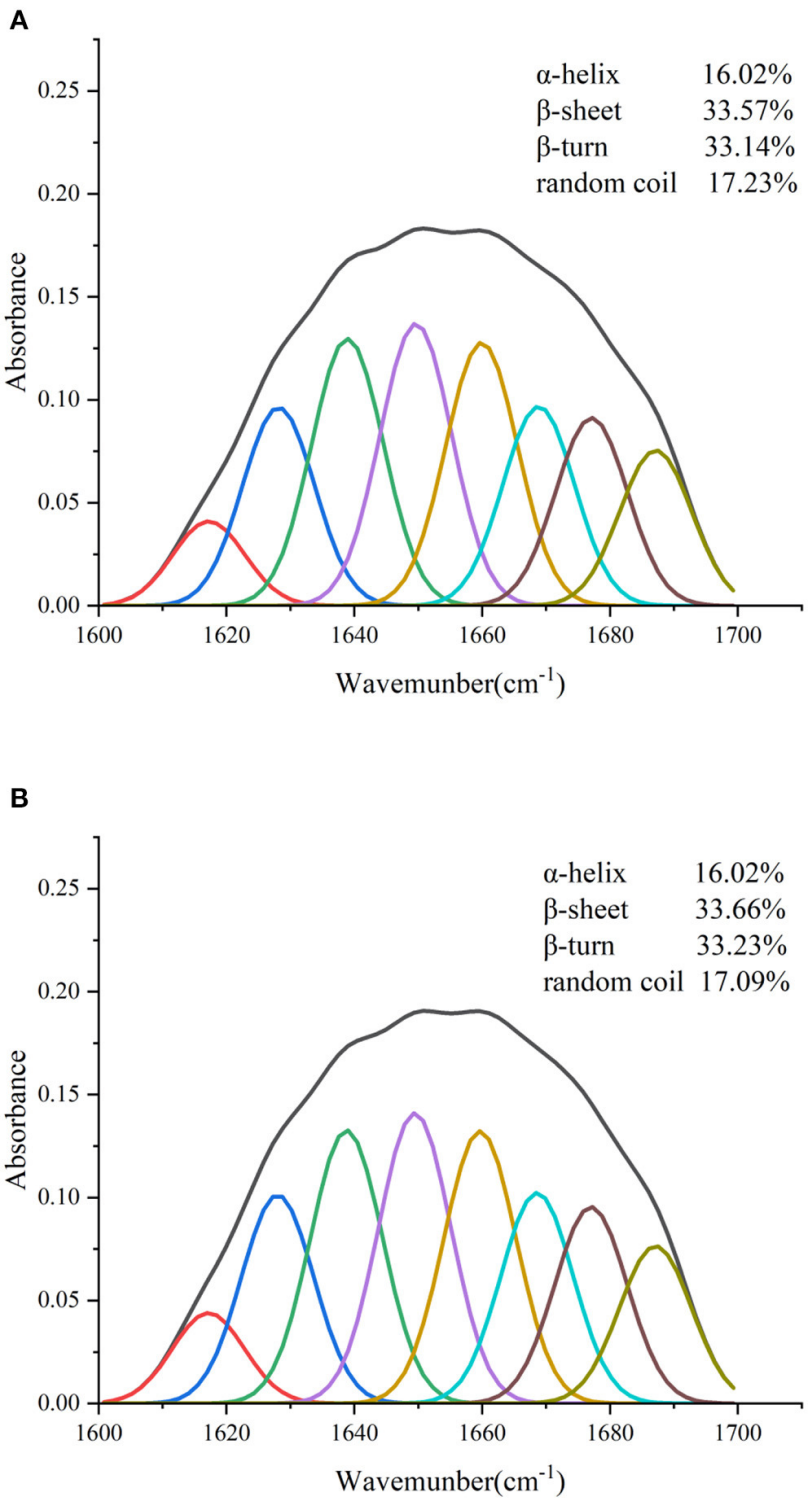
Fourier transform infrared spectrogram of GSPI and GSPI–lutein in the region of 1,600–1,700 cm^−1^. **(A)** GSPI and **(B)** GSPI–lutein.

The combined effect of lutein on the structural changes of GSPI was studied by an FTIR. GSPI was used as a control. As described in Section 3.1.2, the amide I region was the most sensitive region related to protein conformation. The amide I region in the FTIR spectra of GSPI and GSPI–lutein was analyzed, and the secondary structural data were obtained by calculating the integrated area of each sub-peak in the 1,600–1,700 cm^−1^ spectrum. The ratio of each secondary structure of GSPI and GSPI–lutein is depicted in Figure 6. The FTIR spectra indicated that GSPI–lutein was bound through hydrophobic interaction and had little effect on its secondary structure. These results were consistent with the previous study results reporting that SPIHs interacting with cyanin-3-O-glucoside (Cy3G) (60) and SPI (unheated and heated) combined with curcumin (69) had no significant effect on the secondary structure of the protein.

### Effect of GSPI on the Stability of Lutein

#### Color Analysis

**Figure 7:**
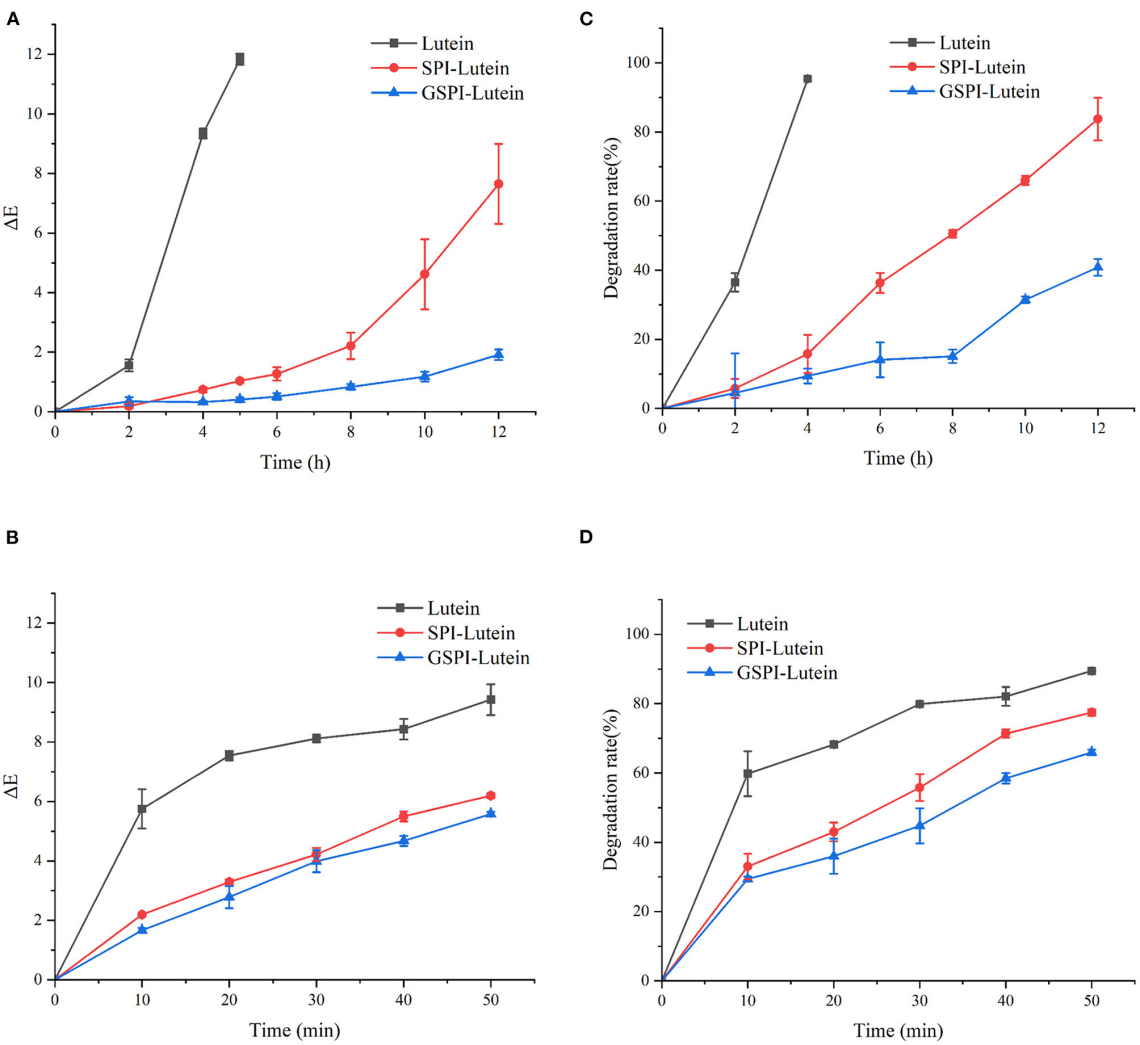
Stability analysis of lutein, SPI–lutein and GSPI–lutein. **(A)** Total color difference under light conditions. **(B)** Total color difference under heating conditions. **(C)** Degradation rate under light conditions. **(D)** Degradation rate under heating conditions.

In addition to physiological functions, lutein is also popular as a coloring agent. Therefore, studying its color changes has high significance. The smaller the total color difference (ΔE), the more stable the lutein (70). ΔE > 3 indicated a very significant difference, 1.5 < ΔE < 3 indicated a significant difference, ΔE < 1.5 indicated a small difference (71). As depicted in Figure 7A, ΔE of lutein reached 11.83 ± 0.18 at 5 h of light exposure. At this time, the ΔE of SPI–lutein and GSPI–lutein was lower, accounting for 1.04 ± 0.06 and 0.41 ± 0.74, respectively. This result indicated that protein had a positive effect on the photostability of lutein. The difference between SPI–lutein and GSPI–lutein appeared with the prolongation of light time. When the light time was 12 h, the ΔE of SPI–lutein and GSPI–lutein was significantly different, accounting for 7.65 ± 1.34 and 1.91 ± 0.18, respectively. GSPI–lutein exhibited better light stability than lutein and SPI–lutein. However, the advantage of thermal stability was not good as light stability (Figure 7B). At 120°C oil bath for 50 min, the ΔE of lutein was 9.42 ± 0.52, the ΔE of SPI–lutein was 6.20 ± 0.08, and the ΔE of GSPI–lutein was 5.58 ± 0.08. Overall, GSPI had a better effect on the color stability of lutein than SPI.

#### Degradation Rate

Lutein is sensitive to environmental factors, such as light and heat, due to its high degree of unsaturation. Figure 7C depicts the degradation rates of lutein, SPI–lutein, and GSPI–lutein under the influence of light and heat, respectively. The degradation rate and total color difference showed the same trend. When lutein was exposed to light for 4 h, the degradation rate reached as high as 95.41% ± 0.34. At this time, the degradation rates of SPI–lutein and GSPI–lutein were 15.78% ± 5.56 and 9.40% ± 2.13, respectively. Compared with lutein, the degradation rate of GSPI–lutein was reduced by about 86.01%. Lutein was completely degraded at 5 h (not shown). With the increase in light time, the degradation rate of GSPI–lutein was always lower than SPI–lutein. Thermal degradation for 10 min showed a significant protective effect of the protein, but there was no significant difference in the protective effect of SPI and GSPI. After 50 min, the degradation rate of lutein was 89.46% ± 0.21, the degradation rate of SPI–lutein was 77.48% ± 0.94, and the degradation rate of GSPI–lutein was 65.96% ± 0.82 (Figure 7D). Overall, GSPI–lutein has excellent light stability and fine stability at high temperatures and short time. Additionally, lutein (with or without protein) followed the first-order degradation kinetics at 120°C and zero-order degradation kinetics under UV light at 25°C (Table 3).

**Table 3:** Correlation coefficients of zero-order, first-order, and second-order kinetic models for the lutein degradation.

**Correlation coefficient**	***R*_0_**	***R*_1_**	***R*_2_**
120°C	0.9676	0.9923	0.9792
25°C UV light	0.9826	0.9677	0.9477

#### Particle Size Analysis

**Figure 8:**
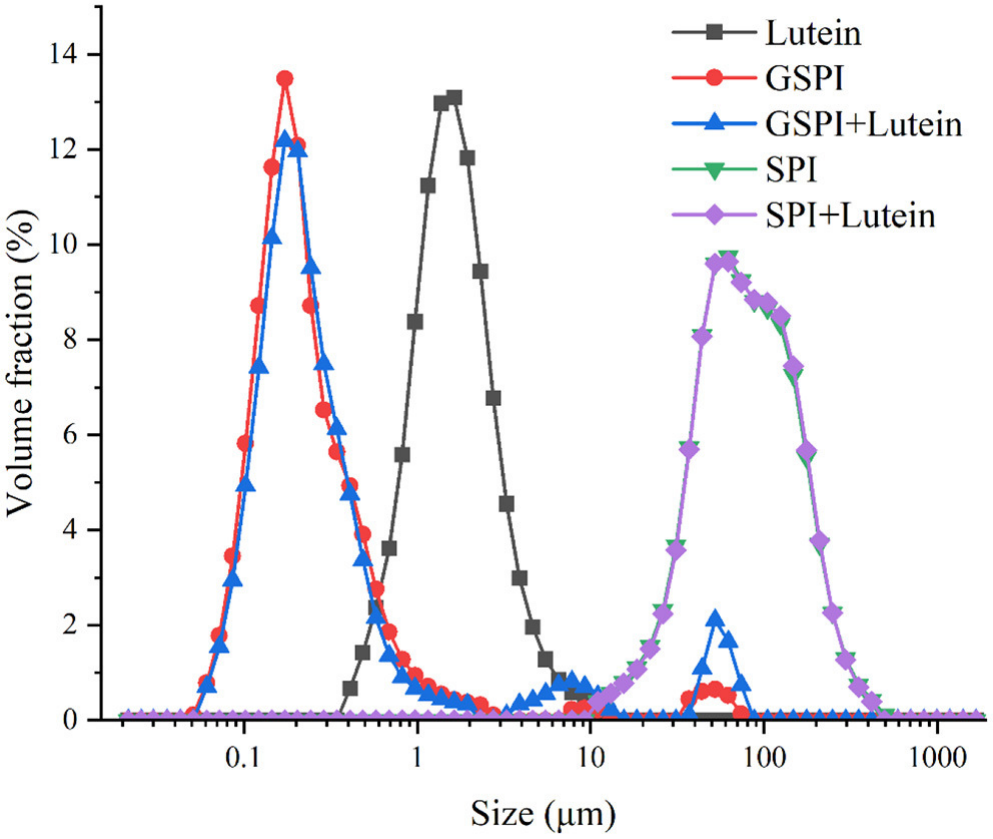
Particle size distribution of lutein, GSPI, GSPI–lutein, SPI, and SPI–lutein.

The particle size affects the stability of the lutein system (32, 72). The smaller the particle size, the greater the stability in the aqueous solution. As depicted in Figure 8, the average particle size of lutein in the aqueous solution was about 2 μm. The average particle sizes of SPI and SPI–lutein were about 83.34 and 84.15 μm, respectively. The average particle sizes of GSPI and GSPI–lutein were about 212 and 219 nm, respectively. GSPI and GSPI–lutein had lower particle sizes, indicating that they were more stable in the aqueous solutions.

## Conclusion

In this study, the effects of glycosylation on the structure and stability of SPI were investigated by fluorescence spectroscopy, FTIR, emulsification, and solubility. The results showed that the glycosylation reaction occurred, and GSPI had better emulsification and solubility than SPI. Then, the interaction between GSPI and lutein was investigated by fluorescence quenching, thermodynamic binding parameters, UV–Vis, and XRD. The results indicated that lutein was spontaneously bound to GSPI at a stoichiometric of 1:1. The fluorescence of GSPI was quenched by static quenching during the binding process, which slightly affected the secondary structure of GSPI. Finally, the total color difference and degradation rate results showed that GSPI had a better stabilization effect on lutein.

## Data Availability Statement

The datasets presented in this study can be found in online repositories. The names of the repository/repositories and accession number(s) can be found in the article/supplementary material.

## Author Contributions

XW: data management, investigation, validation, and writing—original draft. SW: methodology, project management, and writing—review and editing. DX: fund acquisition and supervision. JP: resources, concepts, and supervision. WG: project management, resources, and capital acquisition. YC: conceptualization, project management, and supervision. All authors contributed to the article and approved the submitted version.

## Funding

This research was supported by the National Natural Science Foundation of China (31871808), the School Level Cultivation Fund of Beijing Technology and Business University for Distinguished and Excellent Young Scholars (BTBUYP2020), the Construction of Service Capability of Scientific and Technological Innovation (PXM2019_014213_000010, PXM2018_014213_000033, PXM2018_014213_000014, PXM2018_0142 13_000041, and 19005857058), and the Cultivation and Development of Innovation Base (Z171100002217019).

## Conflict of Interest

JP and WG were employed by Chenguang Biotech Group Co., Ltd. The remaining authors declare that the research was conducted in the absence of any commercial or financial relationships that could be construed as a potential conflict of interest.

## Publisher's Note

All claims expressed in this article are solely those of the authors and do not necessarily represent those of their affiliated organizations, or those of the publisher, the editors and the reviewers. Any product that may be evaluated in this article, or claim that may be made by its manufacturer, is not guaranteed or endorsed by the publisher.
